# Mechanisms underlying the activation of *TERT* transcription and telomerase activity in human cancer: old actors and new players

**DOI:** 10.1038/s41388-019-0872-9

**Published:** 2019-07-08

**Authors:** Xiaotian Yuan, Catharina Larsson, Dawei Xu

**Affiliations:** 1 School of Medicine, Shandong University, 250012 Jinan, People’s Republic of China; 2 Department of Medicine, Center for Molecular Medicine (CMM) and Bioclinicum, Karolinska Institutet and Karolinska University Hospital Solna, 171 64 Solna, Sweden; 3 Department of Oncology-Pathology and Bioclinicum, Karolinska Institutet and Karolinska University Hospital Solna, 171 64 Solna, Sweden

**Keywords:** Oncogenes, Cancer genetics

## Abstract

Long-lived species Homo sapiens have evolved robust protection mechanisms against cancer by repressing telomerase and maintaining short telomeres, thereby delaying the onset of the majority of cancer types until post-reproductive age. Indeed, telomerase is silent in most differentiated human cells, predominantly due to the transcriptional repression of its catalytic component *telomerase reverse transcriptase (TERT)* gene. The lack of telomerase/TERT expression leads to progressive telomere erosion in dividing human cells, whereas critically shortened telomere length induces a permanent growth arrest stage named replicative senescence. TERT/telomerase activation has been experimentally shown to be essential to cellular immortalization and malignant transformation by stabilizing telomere length and erasing the senescence barrier. Consistently, TERT expression/telomerase activity is detectable in up to 90% of human primary cancers. Compelling evidence has also accumulated that TERT contributes to cancer development and progression via multiple activities beyond its canonical telomere-lengthening function. Given these key roles of telomerase and TERT in oncogenesis, great efforts have been made to decipher mechanisms underlying telomerase activation and TERT induction. In the last two decades since the *TERT* gene and promoter were cloned, the derepression of the *TERT* gene has been shown to be achieved typically at a transcriptional level through dysregulation of oncogenic factors or signaling, post-transcriptional/translational regulation and genomic amplification. However, advances in high-throughput next-generation sequencing technologies have prompted a revolution in cancer genomics, which leads to the recent discovery that genomic alterations take center stage in activating the *TERT* gene. In this review article, we summarize critical mechanisms activating *TERT* transcription, with special emphases on the contribution of TERT promoter mutations and structural alterations at the *TERT* locus, and briefly discuss the underlying implications of these genomic events-driven TERT hyperactivity in cancer initiation/progression and potential clinical applications as well.

## Introduction

Cancer formation/progression results from the accumulation of genetic mutations in cells [1]. Because every cell is exposed to mutagens with each round of cell division to a similar extent, and larger animals undergo more cell divisions and experience more mutagenic exposure, the risk of developing cancer should theoretically be higher in species with bigger body sizes and longer lifespan [2]. However, despite 1000-fold more cells and/>30-fold longer lifespan in the human than in the mouse, human cancer risk is actually much lower: Approximately one-quarter of aged people die of cancer, whereas this number can reach up to 90% in aged mice [2]. Several lines of evidence have suggested that naturally occurring strategies against cancer have evolved in long-lived and large-bodied mammalian species, thereby delaying the onset of cancer until post-reproductive age [2]. For instance, large mammalian species including humans (with body mass >5–10 kg) acquire a strong cancer-protection means by repressing telomerase, a ribonucleoprotein enzyme that catalyzes telomeric DNA lengthening on chromosome termini [2, 3]. In contrast, mouse somatic cells are TERT/telomerase-proficient. Moreover, most laboratory mice carry 5–10-fold longer telomere than do humans [3]. Telomerase repression coupled with short telomeres in human cells is believed to confer them a potent barrier to transformation. In support of this view, Hahn et al. [4] experimentally demonstrated that two oncogenes (the simian virus 40 large-T oncoprotein and an oncogenic allele of H-ras) in combination with telomerase activation through ectopic TERT expression are required to directly convert human epithelial cells and fibroblasts into malignant cells, whereas these two oncogenes are sufficient to transform rodent cells into tumorigenic cells without introducing ectopic TERT.

Mechanistically, this telomerase repression and/or shorter telomeres in human cells function to prevent uncontrolled cellular proliferation [2]. The lack of telomerase leads to progressive telomere erosion in dividing human cells due to the intrinsic feature of DNA polymerase [5]. When telomere length shortens to a critical size and telomeres become dysfunctional, the DNA damage response pathway is activated and cells are triggered to enter into a permanent growth arrest stage named replicative senescence. Senescence is believed to act as a very effective barrier against cancer by blocking proliferation and genetic mutations resulting from DNA replication. As infinite proliferation is a hallmark of malignant cells [6], conceivably, overcoming the senescence barrier by telomere stabilization is required in oncogenesis, and, in most cases, this is achieved via telomerase activation [5, 6].

Telomerase is a multi-unit complex, but its core enzyme is only composed of a catalytic component TERT and internal telomerase RNA template (TERC) [5]. TERC is ubiquitously expressed in various human cells while the *TERT* gene is stringently repressed in most human somatic cells, which consequently results in telomerase silencing. Thus, TERT is a rate-limiting determinant for controlling telomerase activity. Indeed, numerous studies have unequivocally demonstrated that TERT induction is required for human cells to acquire telomerase activity. Bodnar et al. [7] elegantly showed that the ectopic introduction of TERT into telomerase-negative human fibroblasts induces telomerase activity, thereby leading to telomere lengthening and immortalization.

**Fig. 1:**
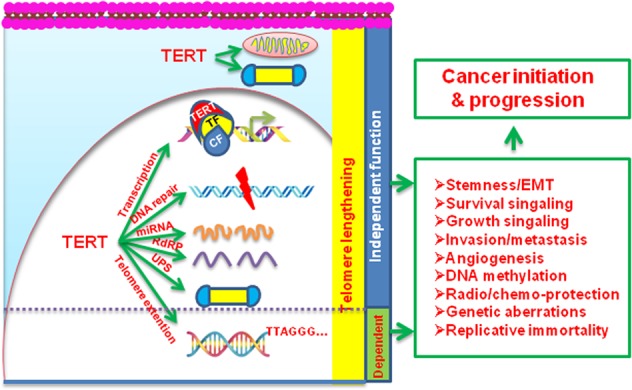
Multiple oncogeneic roles for TERT in cancer development and progression. TERT/telomerase activation is required for transformation of human cells and infinite proliferation by stabilizing telomere length (Telomere lengthening-dependent). The telomere lengthening-independent functions of TERT significantly contribute to cancer initiation/progression, which include its effects on mitochondria, ubiquitin-proteasomal system (UPS), gene transcription, microRNA (miRNA) expression, DNA damage repair, RNA-dependent RNA polymerase (RdRP) activity. CF co-factor; EMT epithelial-mesenchymal transition, TF transcription factor. The effect of TERT on UPS predominantly occurs in the nucleus, but is also possible in the cytoplasma

TERT induction/telomerase activation confers unlimited proliferation potential to cancer cells by stabilizing their telomere length, while recent observations reveal its multiple oncogenic activities independently of a telomere-lengthening function, which include its effect on mitochondrial and ubiquitin-proteasomal function, DNA damage repair, gene transcription, microRNA expression, etc. [8–16]. Importantly, TERT was previously shown to promote the proliferation of normal mouse stem cells by recruiting chromatin-remodeling factor Brg1 to β-catenin target genes for their transcriptional activation [17]; and more recently, TERT was found to directly interact with β-catenin and robustly amplify its transcriptional outputs, thereby stimulating epithelial mesenchymal transformation (EMT) and stemness of cancer cells [12, 13]. TERT is also required for symmetric stem cell division and its high expression significantly increases cancer stem cell (CSC) pool and self-renewal in prostate cancer [13]. It has also been revealed that TERT displays a RNA-dependent RNA polymerase (RdRP) activity, thereby regulating mitotic progression and cancer stem cell traits [18]. In addition, TERT interacts with NF-κb p65, activating NF-κb target genes and upregulating the expression of a number of metalloproteinases (MMPs) in cancer cells [14]. The enhanced EMT, stemness, and MMP expression all contribute to invasiveness and metastasis in cancer, which indicates an important role of TERT in cancer progression. Liu et al. further demonstrated that TERT served as a partner for the transcription factor Sp1 to facilitate cancer angiogenesis [19]. We recently found that this same mechanism was involved in TERT-mediated aberrant DNA methylation and silencing of tumor suppressor genes in hepatocellular cell carcinoma (HCC) cells [20]. Taken together, TERT or telomerase may contribute to multi-cancer hallmarks via its telomere lengthening-dependent and independent functions (Fig. 1).

The evolutionarily acquired telomerase repression as a protective strategy against cancer, together with the multiple oncogenic effects of TERT/telomerase as revealed by modern experimental approaches, have pointed to the importance of TERT/telomerase in cancer development and progression. Therefore, great efforts have been made to dissect how the *TERT* gene is transcriptionally de-repressed and telomerase is activated during oncogenesis. Here, we present an overview of the mechanistic insights into cancer-specific TERT expression and biological/clinical implications, paying special attentions to the contribution of genomic aberrations to the *TERT* trans-activation.

### The *TERT* gene, and its promoter and transcripts

**Fig. 2:**
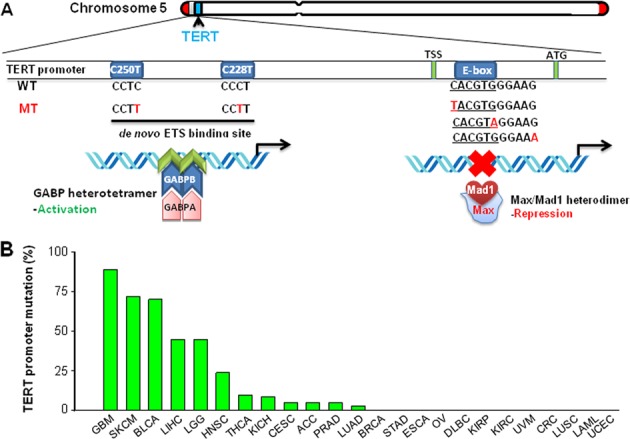
TERT promoter mutations in human cancer. **a** Schematic presentation of TERT promoter mutations and relevant transcription factors. The *TERT* gene at chromosome 5p and its promoter is shown. C > T mutation occurs at one of both positions of the TERT proximal promoter (−124 and −146 to ATG for C228T and C250T, respectively) in malignant cells, which create de novo ETS binding motifs. The ETS family members GABPA and GABPB form heterotetramers that bind to the de novo ETS site and activate *TERT* transcription. The E-box (CACGTG) sequence mutation was recently identified in clear cell renal cell carcinoma (ccRCC), which may lead to the dissociation of the repressor MAX/Mad1 complex from E-box, thereby de-repressing the *TERT* gene. **b** The frequency of TERT promoter mutations in a panel cancer types from the TCGA dataset analyses [49]. GBM glioblastoma multiforme, SKCM skin cutaneous melanoma, BLCA bladder urothelial carcinoma, LIHC liver hepatocellular carcinoma, LGG brain lower-grade glioma, HNSC head and neck squamous cell carcinoma, THCA thyroid carcinoma, KICH kidney chromophobe, CESC cervical squamous cell carcinoma and endocervical adenocarcinoma, ACC adrenocortical carcinoma, PRAD prostate adenocarcinoma, LUAD lung adenocarcinoma, BRCA breast invasive carcinoma, STAD stomach adenocarcinoma, ESCA esophageal carcinoma, OV ovarian serous cystadenocarcinoma, DLBC lymphoid neoplasm diffuse large B cell lymphoma, KIRP kidney renal papillary cell carcinoma, KIRC kidney renal clear cell carcinoma, UVM Uveal melanoma, SARC sarcoma, CRC colorectal carcinoma, LAML acute myeloid leukemia, LUSC lung squamous cell carcinoma, UCEC uterine corpus endometrial carcinoma

The *TERT* gene consists of 16 exons and 15 introns within a ~40 kb gene body and is localized on the short arm of the chromosome 5 (5p.15:33), a megabase distance from the 5p end (Fig. 2) [21, 22]. The *TERT* harbors a single promoter embedded in a CpG island (−1800 to +2300 relative to ATG), while the proximal core promoter is within a 330 bp upstream and 37 bp downstream from ATG (−330 to +37) [23–26]. Remarkably, the TERT promoter region is rich with biding motifs for multiple transcription factors including the MYC oncogene (E-box) and Sp1 (GC box), but lack TATA and CAAT boxes [21, 27]. On the other hand, the promoter also carries sites for repressor binding. Another unique feature is that the TERT promoter is unmethylated in normal human cells, whereas methylated in malignant cells, and Lee et al. identified the 52 CpG-containing TERT hypermethylated oncological region (THOR) as a cancer-associated epigenetic mechanism of TERT upregulation [23–26]. Several lines of evidence suggest that the unmethylated promoter sequence favors a repressor-binding [23].

The *TERT* is a single copy gene with a single transcription start site, but subject to alternatively splicing regulation [21, 28]. More than 20 splicing variants have been identified, while the full-length TERT mRNA is the only one that translates into a functional protein for telomerase activity [28]. Some of these spliced variants such as α-variants, β-variants, and γ-variants miss parts of sequences encoding the reverse transcriptase domain, and may therefore exert a dominant negative effect [28]. Intriguingly, certain normal human cells have been observed to express non-functional transcripts [28–30], but the underlying physiological significance remains unclear.

### Aberrant *TERT* transcription in human cancer: dysregulated positive and/or negative players

The cloning of the TERT promoter and identification of its binding motifs for various transcription factors has contributed to profound insights into TERT/telomerase regulation in human cells [21, 27]. The early studies showed that TERT promoter activity correlated closely with TERT mRNA expression: significantly higher in telomerase-positive cancer cells than in telomerase-negative normal ones, which suggests that controlling TERT expression at a transcriptional level represents a fundamental mechanism to activate telomerase in cancer cells [21, 27].

The findings accumulated in the last 20 years have shown that the transcriptional regulation of the *TERT* gene occurs at multiple levels by various positive and negative factors or signaling pathways. The TERT promoter is bound by different transcription factors, responds to numerous signals and integrates these diverse and dynamic inputs to set the TERT expression output [31]. Furthermore, epigenetic effects on chromatin structure and remodeling of the TERT promoter region add another layer controlling the *TERT* transcription [31]. In addition, many factors indirectly regulate *TERT* transcription by cooperating with transcription factors or other regulatory elements in a context-dependent or independent manner. All these regulators coordinately and tightly control the *TERT* gene to ensure its silence in most normal cells, while its expression at the right time, right place and right quantity only in a small subset of cells, such as activated lymphocytes and stem/progenitor cells [31]. However, this tightly regulated balance is readily disrupted in malignant cells, most likely due to aberrant expression of positive regulators or silencing/sequestering of negative ones [31]. The most typical example are the Myc/Max/Mad1 network proteins [31–33]. MYC is the first identified cellular oncogene to activate telomerase [34]. Ectopic expression of c-MYC in human fibroblasts or epithelial cells robustly induced TERT expression and telomerase activity [34]. In leukemic HL60 cells, a high level of c-Myc expression is coupled with its binding to the E-Boxes on the TERT proximal promoter and TERT mRNA abundance. Once HL60 cells are induced to undergo terminal differentiation, c-Myc expression is diminished, whereas Mad1 levels increase and it consequently replace c-MYC on the TERT promoter, repressing *TERT* transcription [32]. Casillas et al. determined the *TERT* gene trans-activation by endogenous c-Myc during the transformation process of human fibroblasts, and they observed that the endogenous c-Myc expression resulted in a switch from Mad1/Max to c-Myc/Max binding to E-boxes on the TERT promoter, TERT expression and telomerase activation [33]. These changes at the TERT promoter are totally opposite to what happens in differentiated leukemic HL60 cells [32]. In addition, many other factors regulate TERT transcription through the Myc/Max/Mad protein family or different mechanisms. These regulators include the TGF-β/Smad signaling pathway, endoplasmic reticulum stress, NFX1 Tax, estrogen, Ets, DJ-1, E2F, survivin, HIFs, FoxM1, Reptin, Wnt/β-Catenin, Arsenic, Aurora-A, cold inducible RNA-binding protein, various growth factors, and cytokines, etc. [31, 35–44].

The presence of transcription factors is essential to the *TERT* transcription regulation. However, gene transcription involves not only the assembly of transcription factors at promoter/enhancer regions, but also the regulation of accessibility to DNA, a process controlled by an epigenetic mechanism [31, 32, 42, 45]. Thus, epigenetic factors are another group of proteins that modulate *TERT* transcription. DNA methylation, histone acetylation, methylation, and phosphorylation have all been shown to be involved in the *TERT* transcription regulation [31, 32, 42, 45, 46]. The TERT promoter is in general unmethylated in normal cells, while its methylation is required for TERT expression and telomerase activation in cancer cells [23–26]. Histone acetylation/deacetylation was shown to be a common underlying feature to *TERT* transactivation/repression in human cells [31, 32, 42, 45]. Mechanistically, transcription factors Myc/Max/Mad and Sp1 interact with and recruit histone acetyltransferases (HATs) or histone deacetylases (HDACs) to the TERT promoter, dependent on the promoter status and cellular contexts [31, 32, 42]. In addition, SMYD3 as a histone methytransferase is capable of directly binding to CCCTCC sequences on the TERT promoter and specifically catalyzes H3-K4 tri-methylation, through which *TERT* transcription is activated. SMYD3-mediated H3-K4 tri-methylation is required for optimal occupancy of c-MYC and Sp1 on the TERT promoter [45]. Thus, epigenetic factors intimately cross-talk and cooperate with transcription factors to exert their regulatory effects on *TERT* transcription.

In addition to the endogenous TERT regulators discussed above, certain tumor viruses encode proteins that stimulate *TERT* transcription. These exogenous regulators include Epstein-Barr virus (EBV), cytomegalovirus (CMV), Kaposi sarcoma-associated herpesvirus (KSHV), human papillomavirus (HPV), hepatitis B virus (HBV), hepatitis C virus (HCV), human T-cell leukemia virus-1 (HTLV-1), and others [47, 48]. The HPV E6 is the most extensively studied viral oncoprotein for its role in the *TERT* transcription. E6 forms a tertiary complex with E6AP and c-Myc, and such complex then binds to the E-box in the TERT core promoter and subsequently induces promoter activation [47]. The CMV early protein 72 robustly activates *TERT* transcription by interacting with Sp1 [48]. Thus, the targeted activation of *TERT* transcription is one of the key mechanisms for virus-mediated carcinogenesis.

### Aberrant *TERT* transcription in human cancer: genomic alterations as new players at center stage

**Fig. 3:**
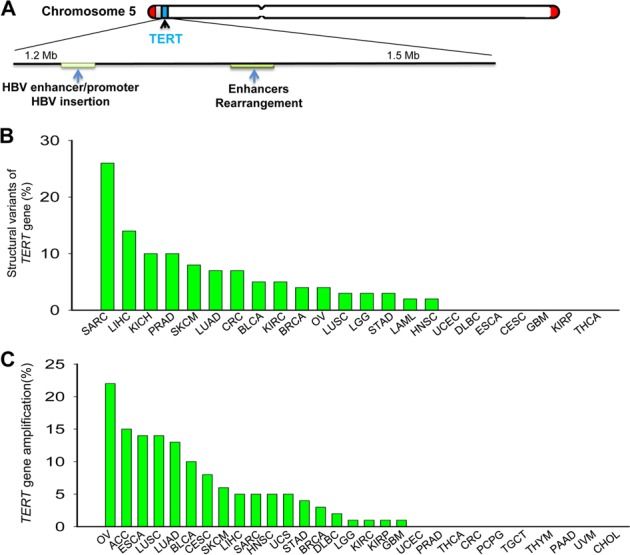
The structural alterations and amplification of the *TERT* gene in human cancer. **a** Schematic presentation of rearrangements and onco-viral insertation of the *TERT* locus. Left: The rearrangements occur most frequently in a 50 kb region proximal of the *TERT* gene, although the translocation to other chromsomes is also observed. The rearrangements are not random events, which often juxtapose the TERT coding sequence to strong enhancer elements. Right: Most integration breakpoints for onco-viral insertions are located in the TERT promoter region, and almost all the integrations contained at least one viral gene enhancer or promoter. **b** and **c** The structural variants and amplification of the *TERT* gene in a panel of human malignancies from the TCGA cohort analyses, respectively. GBM glioblastoma multiforme, SKCM skin cutaneous melanoma, BLCA bladder urothelial carcinoma, LIHC liver hepatocellular carcinoma, LGG brain lower-grade glioma, HNSC head and neck squamous cell carcinoma, THCA thyroid carcinoma, CESC cervical squamous cell carcinoma and endocervical adenocarcinoma, ACC adrenocortical carcinoma, PRAD prostate adenocarcinoma, LUSC lung squamous cell carcinoma, LUAD lung adenocarcinoma, PCPG Pheochromocytoma and paraganglioma, LUSC lung squamous cell carcinoma, TGCT testicular germ cell tumor, BRCA breast invasive carcinoma, STAD stomach adenocarcinoma, ESCA esophageal carcinoma, OV ovarian serous cystadenocarcinoma, DLBC lymphoid neoplasm diffuse large B cell lymphoma, KIRP kidney renal papillary cell carcinoma, KIRC kidney renal clear cell carcinoma, KICH kidney chromophobe, UVM uveal melanoma, SARC sarcoma, THYM thymus, CRC colorectal carcinoma, LAML acute myeloid leukemia, UCEC uterine corpus endometrial carcinoma, CHOL cholangiocarcinoma

Advances in high-throughput sequencing technologies have enabled comprehensive genomic characterization of various human malignancies and numerous genomic aberrations have been unraveled. Barthel et al. [49] recently examined telomere length, TERT expression and related genomic alterations in 31 cancer types derived from The Cancer Genome Atlas (TCGA) cohort of patients, and they found that the TERT promoter mutations, and focal amplification/rearrangements were intimately associated with acquisition of TERT expression and telomerase activity in tumors (Figs. 2 and 3). These analysis results, together with recent findings by others, suggest that genomic aberrations act as pivotal players in the activation of the *TERT* transcription.

### The recurrent TERT promoter mutations in human cancer: gain-of-function

TERT promoter mutations occur predominantly at two hotspots of chromosome 5 (1,295,228 and 1,295,250, or −124 and −146 bp from the ATG) with a cytidine-to-thymidine (C > T) dipyrimidine transition, thus named C228T and C250T, respectively (Fig. 2a). These mutations, originally identified in sporadic and familiar malignant melanomas in 2013 [50, 51], have since then been widely investigated and observed in various types of human cancer with different frequencies (summarized in details in recent reviews [31, 52]). Glioblastoma, malignant melanoma, urothelial bladder cancer, myxoid liposarcoma, and certain subtypes of skin cancer and medulloblastoma exhibit the highest TERT promoter mutation rate (up to 80–90%). The intermediate level of the mutation frequency (15–50%) is observed in thyroid cancer, hepatocellular carcinoma (HCC), upper tract urinary carcinoma (UTUC), head and neck cancer, ovarian clear cell carcinoma and among others (Fig. 2). Whereas the rest of examined tumor types (lung, breast, gastrointestinal, prostate and kidney cancer, and almost all hematological malignancies) lack the mutations or have a low mutation rate (<10%). An average of 27% tumors was observed to bear TERT promoter mutations in the TCGA pooled cohort, and they have so far been the most common mutations identified in non-coding regulatory regions in human cancer [49].

The C228T mutation is more prevalent than C250T among various malignancies and their presence is mutually exclusive, which suggests their functional redundancy. Indeed, primary tumors bearing either mutation tend to express higher levels of TERT mRNA and telomerase activity, implying a stimulatory effect on TERT expression [31, 49]. Creating a C228T mutation in the TERT promoter region using a CRISPR technique in human pluripotent stem cells, Chiba et al. found that these cells constitutively expressed TERT and telomerase even after having undergone terminal differentiation, in sharp contrast to the wild-type (wt) TERT promoter-bearing stem cell-derived progenies where the *TERT* transcription was shut down following cellular differentiation [53]. Moreover, the differentiated cells with mutant TERT promoter carried longer telomeres and erased replicative senescence imposed by telomere attrition as seen in normal cells [53]. Similarly, Li et al. introduced the C228T mutation into the TERT promoter in normal human bladder stem cells and this single event was sufficient to drive transformation of these stem cells [54]. Thus, their findings provide direct evidence that the presence of C228T or C250T mutation confers cells immortality or sustained proliferation potential and promotes their transformation by activating telomerase.

To determine how these two mutations upregulate TERT expression, Huang et al. [51] generated C228T-bearing or C250T-bearing TERT promoter reporter constructs and observed a robust increase of the promoter activity, suggesting a gain-of-function effect on the *TERT* transcription. It has now been clear that these mutations create a de novo binding site for ETS transcription factors [51, 55]. Bell et al. and Mancini et al. further showed that GA-binding proteins (GABPA and GABPB1), the members of the ETS family transcription factors, were specifically recruited to the mutant rather than wt TERT promoter in cancer cells, thereby activating *TERT* transcription and telomerase (Fig. 2) [55, 56]. Consistently, inhibiting the expression of GABPA or GABPB1 rather than other ETS members led to diminished *TERT* expression in cancer cells bearing a mutant TERT promoter [55, 56]. In cancer cells carrying heterozygous TERT promoter mutations, the mutant promoter recruits GABPA and exhibits the H3K4me2/3 mark of active chromatin [57]. In contrast, the wild-type allele retains the H3K27me3 mark of epigenetic silencing [57]. These results suggest that only the mutant promoters are transcriptionally active. Moreover, GABPA needs to form a heterotetramer complex [(GABPA/GABPB)_2_] with its partner GABPB1 or GABPB2 through which DNA binding and transcriptional regulation is achieved. Mancini et al. showed that mutant TERT promoter-harboring glioblastoma cells similarly exhibited decreased TERT expression upon GABPB1 knockdown, and these GABPB1-depleted cells also suffered impaired proliferation/survival, telomere shortening/dysfunction and attenuated tumorigenic ability [56]. Given these findings, together with previously observed oncogenic effects on prostate, breast cancer and leukemia [58], GABPA/B1 has recently been suggested as a novel therapeutic target for tumors harboring a mutant TERT promoter [56].

Surprisingly, however, our results obtained from thyroid cancer (TC) studies question it as a therapeutic target [59]. First, GABPA depletion leads to significant downregulation of TERT expression in TC-derived cells independently of TERT promoter mutations; Second, GABPA expression is negatively correlated with TERT expression in primary tumors from TC patients; Third, GABPA depletion robustly increases thyroid cancer cell invasion despite a diminished TERT expression. Mechanistically, we identified *DICER1*, a component of the microRNA machinery to inhibit cancer metastasis, as a direct target gene for GABPA. By promoting DICER1 expression, GABPA inhibits the invasive phenotype of thyroid cancer cells. Forth, lower rather than higher GABPA expression is positively correlated with aggressive disease and poor outcomes in TC patients. Finally, knocking-down GABPB1 expression in TC cells similarly facilitated invasiveness while inhibited TERT expression (Unpublished data). These observations raise a number of critical questions: How does GABPA regulate TERT expression in wt TERT promoter-carrying TC cells? Whether the effects of GABPA on the wt TERT promoter and cancer development/progression are context-dependent? Whether GABPA could serve as a therapeutic target for cancer? Elucidating these issues should be both biologically and clinically important.

In addition to C228T and C250T mutations, CC > TT tandem mutations at position −124/−125 bp and −138/−139 (from ATG) were found in a subset of cancer [31, 60]. These two tandem mutations also lead to the creation of the ETS transcription factor-binding motif [31]. More recently, the mutation at the MYC binding site in the TERT promoter was documented in 8% of tumors from patients with clear cell renal cell carcinoma (ccRCC) (Fig. 2a) [61]. It is suggested that this type of the mutation may prevent the binding by repressors in the MYC network family, thereby de-repressing the *TERT* transcription [61]. Tumors carrying these mutations have the longest telomere compared to those with wt and C228T/C250T-TERT promoters [61]. However, the exact functional implication of this genetic alteration is unclear, and it is also unknown if this mutation is unique to ccRCC or present in other cancers, which calls for further investigations.

The TERT promoter mutation rate varies significantly from undetectable to more than 90% among studied human malignancies, and it remains poorly understood what cause such differential mutation distributions among different types of cancer [31]. We as well as others found that the presence of TERT promoter mutations was negatively correlated with telomere length in tumors [31, 62]. Moreover, senior age is also intimately associated with the mutation occurrence [62]; because progressive telomere erosion occurs with increased age, this observed age-mutation link is likely due to shorter telomeres. It is well established that dysfunctional telomeres trigger genomic instability [5, 63], and conceivably, shortened telomeres may be a key driving-force for the onset of the TERT promoter mutation during malignant transformation. This view is strongly supported by a recent report from Garcia’s group. They observed that the spontaneous acquisition of *TERT* promoter mutations was selected for in blood cells from non-tumor individuals with very short telomere due to germline defects in the TERT protein or other telomerase components [64]. We found that the germline variants at the *TERT* locus significantly affected the TERT promoter mutation rate in HCC patients, and the *TERT* rs2736100-CC cancer risk genotype was more frequently seen in patients with a wt TERT promoter [65]. This variant is known to upregulate TERT expression, thereby counteracting telomere shortening [66].

### *TERT* rearrangements and onco-viral DNA insertions: connecting to enhancer hijacking

The cancer genomic landscape mapping has recently identified both rearrangements and oncogenic viral genome insertions at the *TERT* locus as novel mechanisms to upregulate TERT expression by hijacking enhancers (Fig. 3a) [49, 67]. Enhancers, non-coding and long-range cis-regulatory elements, regulate gene transcription by interacting with gene promoters but independently of their position and orientation with respect to the transcription start site [68]. These regulatory elements, marked by monomethylation of histone H3 at lysine 4 and acetylation of histone H3 at lysine 27, bind to different proteins involved in the establishment and maintenance of chromatin loopings [68]. Most enhancer elements are normally inaccessible due to closed nucleosome structure, while a subset of sequence-specific transcription factors can act as pioneer factors and erase this barrier to occupy their specific sequences [68]. These pioneer factors make the underlying DNA more accessible to the transcriptional machinery, thereby promoting the recruitment of a Mediator complex, and facilitating enhancer interaction with the basal transcription machinery and RNA polymerase II at promoters in a gene-specific manner. Moreover, there exist super-enhancers, a small set of enhancers spanning large regions of the genome in a clustered manner, and in cancer cells, super-enhancers are frequently associated with oncogene activation [68].

### The TERT gene rearrangements

Zhao et al. [69] first noticed the rearrangement of the *TERT* locus coupled with TERT induction/telomerase activation in immortalized human fibroblasts. Then the analyses of mantle cell lymphoma (MCL) using multi-color FISH unraveled the *TERT* rearrangement in 4/8 MCL-derived cell lines and 1/23 patients [70]. Importantly, all these cell lines and patient cells expressed significantly higher levels of the *TERT* gene [70]. However, a detailed analysis was not performed in these earlier reports. Recently, using high-throughput sequencing, this genomic event has been identified to activate telomerase in many cancer types, including neuroblastoma, glioblastoma, meningiomas, malignant melanoma and pheochromocytomas, chromophobe renal cell carcinoma (ChRCC), HCC, and among others [71–78] (Fig. 3b). The TCGA cohort patient analyses showed that the structural variants/rearrangements involved in the *TERT* locus were similarly observed and the highest frequency occurred in sarcoma (22%) [49] (Fig. 3b).

Based on the analyses of NIH Epigenomics Roadmap data (containing 127 human tissues), Valentijn et al. [73] documented that the 20-kb region upstream of *TERT* was highly repressed across all investigated tissues and displayed chromatin modifications characteristic of a Polycomb signature, whereas super-enhancers were readily found beyond this repressive region. Thus, the disruption of this Polycomb-silenced region likely leads to *TERT* activation [73]. *TERT* rearrangements are not random events, because the majority of the *TERT* rearrangements lead to the direct overlapping between super-enhancers and juxtaposed *TERT* coding sequence. Such an overlapping causes enhancer-hijacking through which massive chromatin remodeling and transcriptional activation of the *TERT* gene is achieved [73]. The TCGA data analyses and many other observations indeed show that *TERT* rearrangements induce TERT expression much more efficiently than TERT promoter mutations [49].

The *TERT* rearrangement has been most comprehensively dissected in neuroblasoma, a malignancy of the sympathetic nervous system in infants and young children [71–73]. Peifer et al. [71] observed the recurrent genomic rearrangements in a 50 kb region proximal of the *TERT* gene in high risk/stage (III and IV) tumors with a frequency of 23% (39/169) (Fig. 3a). Similar scenarios were also documented in two other publications [72, 73]. Interestingly, the rearrangement event is mutually exclusive with MYCN aberrations and ATRX mutations [71–73]. MYCN is known to activate the *TERT* gene and the *MYCN* amplification is the most frequent genomic alteration in neuroblastoma. ATRX encodes a SWI/SNF chromatin-remodeling ATP-dependent helicase, and its mutation leads to the functional inactivation of the gene product, thereby promoting alternative lengthening of telomeres (ALT), a recombination-based mechanism for telomere maintenance [71–73]. Taken together, the different subgroups of neuroblastoma employ different mechanisms for telomere lengthening and sustained cellular proliferation.

### Onco-viral DNA insertions

Infection of oncogenic virus is responsible for up to 15% of human malignancies [67], and activation of telomerase is one of the key mechanisms behind viral carcinogenesis [47]. It has long been suggested that viral proteins derived from HPV, HBV, EBV, CMV, and others serve as co-factors to activate *TERT* transcription, as described above [47]. Recent evidence has also accumulated that the integration of the viral DNA into the *TERT* locus represents additional novel mechanisms for TERT regulation (Fig. 3a).

HBV infection is intimately associated with HCC development, which provides an ideal study model. The integration of the HBV DNA into host genome has long been realized as a pivotal carcinogenic driving-force, however, underlying mechanisms are elusive. In a study of HCC cell lines, the HBV enhancer-containing DNA fragment was found to insert into the 5’ regulatory region at the 1.6 kb upstream of the *TERT* transcription start site and this exogenous viral enhancer drove endogenous *TERT* transcription in HCC cells [79]. Using a PCR-based assay, Paterlini-Bréchot et al. showed that HBV targeted the *TERT* gene for integration in 2 out 22 HCC tumors [43]. To thoroughly elucidate the role for HBV-host genome interaction in HCC pathogenesis, Sung et al. [80] conducted a massive sequencing analysis of 81 HBV-positive HCC tumors. They found that the *TERT* locus was the most frequent target as HBV integration breakpoints and the insertion of HBV DNA were identified in 18 of these HCC tumors [80]. Most of the integration breakpoints were located in the TERT promoter region, and almost all the integrations contained at least one viral gene enhancer or promoter [67] (Fig. 3a). Consistently, higher levels of TERT expression were readily seen in these tumors [67, 80]. For HBV-negative HCC, adeno-associated virus type 2 (AAV2), a member of the parvovirus group, may be involved in the oncogenic process. Nault et al. [81] showed that AAV2 DNA was integrated into the host genome in 11 of 193 HCC tumors and all the breakpoints affected cancer-related genes including *TERT*. The 208 bp AAV2 DNA fragment, cloned from the patient tumor and when inserted into the TERT promoter reporter construct, significantly increased luciferase activity, suggesting a functional significance of the inserted AAV2 sequence in telomerase activation [81].

The findings described above demonstrate that HCC cells acquire TERT overexpression and telomerase activation by hijacking integrated HBV promoters or enhancers at the *TERT* locus, however, little has been known about the genomic interaction between the host *TERT* and other onco-viruses. Chen et al. screened for the DNA integration from a panel of onco-viruses including HPV, EBV, and BKV in different types of tumors, and they did detect the presence of insertion events, but integration breakpoints were located at other cancer-driving genes rather than the *TERT* locus [67]. However, another recent study showed that HPV DNA targeted the *TERT* locus for integration in cells and tumors derived from head and neck cancer [82], but more detailed and comprehensive analyses are required.

### *TERT* amplification: matter of gene dosages

The amplification of genes encoding oncogenic products frequently occurs in cancer. For example, the *MYCN* amplification is the most common genomic aberration in high-risk neuroblastoma, which leads to MYCN overexpression, driving aggressive diseases [71]. Our earlier study demonstrated that TERT expression was gene-dosage dependent and haploinsufficient for telomere maintenance in human cells [83], suggesting this gene as an amplified target in carcinogenesis. Indeed, we and Keith’s group first identified the *TERT* amplification in human cancer almost 20 years ago [84, 85]. In neuroblastoma cells, we observed that the *TERT* gene was typically amplified in double-minuses, and each cell harbored more than 100 *TERT* copies [84]. In other tumors, focal copy gains or amplification at the *TERT* locus were frequently detected [31, 60, 86]. Numerous studies have since then shown the widespread of this genomic event in many different types of malignancies. Moreover, high-throughput next-generation sequencing has been performed on most human cancer types in the last decade, which have provided rich tumor genomic information. Barthel et al. [49] analyzed the *TERT* gene amplification in the TCGA cohort including 6835 patients and covering 31 tumor types, and the findings are summarized as follow: (i) A total of 4% the examined tumors exhibit a *TERT* gene amplification, with high frequency in ovarian cancer, adrenocortical carcinoma, esophageal cancer, lung adenocarcinoma, and squamous carcinoma (Fig. 3c); (ii) *TERT* amplification is observed in 3% of TERT-expressing tumors; and (iii) The highest telomerase activity is found in tumors with a *TERT* amplification. These results obtained from the TCGA cohort provide a general amplification profile of the *TERT* gene in human cancer, and indicate a key role of this genomic aberration in activation of telomerase during cancer development and/or progression.

### *TERT* transcription via telomere position effect over long distances (TPE-OLD): genomic-epigenetic interaction

Genes near telomeres are regulated by a mechanism that depends both on telomere length and on the distance to the gene, so-called telomere position effect (TPE) [5]. More recently, TERT and some other genes, although localized at positions with a certain distance from telomeres (Figs. 2a and 3a), were also found to be regulated by telomere length, a TPE-like mechanism or TPE-over long distances (TPE-OLD) [22]. In normal young human cells with long telomeres, a telomere-loop structure is formed in the region near the *TERT* locus, leading to a repressed *TER*T epigenetic state, however, shortened telomeres in aged cells disrupt the repressive loop, which consequently opens the closed chromatin structure and induces *TERT* transcription. Because very short telomeres are the most frequent genomic alteration in human tumors, TPE-OLD likely plays a part in TERT/telomerase activation in cancer cells. It is currently unclear whether or how much this mechanism contributes to cancer-related *TERT* transcription, and whether it cooperates with other regulatory cascades to activate telomerase. The elucidation of these issues will gain insights into not only telomere biology but also the relationship between age and cancer.

### Clinical implications/applications in precision oncology

#### Biomarkers for cancer diagnosis and disease surveillance

Cancer-specific TERT expression and telomerase activation has always aroused great enthusiasm for a potential clinical application of TERT/telomerase-based assays in the cancer field. However, a number of problems (such as unstable TERT mRNA and enzymatic activity) impede reliable utility of a direct TERT expression or telomerase activity assay for cancer diagnostic or monitoring purpose, whereas widespread TERT promoter mutations in different tumors pave new avenues [31]. The non-invasive detection of a mutant TERT promoter is especially attractive and has been evaluated in plasma, urine and cerebrospinal fluid (CSF) for the diagnosis/monitoring of HCC, bladder cancer and glioblastoma, respectively [31, 87–90]. These proof-of-concept studies have shown usefulness of the mutant TERT promoter as a non-invasive assay biomarker for these malignancies.

Since many types of human cancer lack any TERT promoter mutations, and even in bladder cancer and glioblastoma, up to 30% of the tumors are negative for a mutation, other telomerase-related markers are apparently required for those patients. The hypermethylated TERT promoter has also been shown to be unique to human cancer as described above, and might serve as a diagnostic biomarker. We have recently identified two methylated CpGs in the TERT promoter region specific to tumors from patients with gastrointestinal cancer (GIC), and the methylated sites were detectable in stool from GIC patients, with sensitivity and specificity comparable to a fecal occult blood test [24]. Bougel et al. found that the methylated TERT promoter detection in CSF could predict leptomeningeal metastasis [91]. These studies suggest the feasibility of *TERT* promoter methylation analyses as an additional tool in noninvasive cancer diagnosis and disease surveillance.

#### Outcome prediction

Numerous clinical studies have evaluated telomerase/TERT-related alterations as prognostic factors for cancer patients. Higher TERT expression in tumors was observed to predict poor patient outcomes in a panel of cancer types [31, 49, 92]. In neuroblastoma, tumors without detectable TERT mRNA frequently undergo spontaneous regression [93], whereas TERT expression, and/or its gene amplification or rearrangements are closely associated with a high-risk/aggressive disease and shorter survival [71–73]. The presence of TERT promoter mutations has recently been shown as a unfavorable prognostic factor in a number of cancer types including papillary thyroid carcinoma, glioblastoma, bladder cancer, and others [31, 94–97]. In addition, the association between TERT promoter hypermehtylation and poor outcomes or progression was reported in brain tumors and adrenocortical carcinoma [25, 98]. Collectively, the aberrant TERT expression and TERT-related genomic alterations may serve as prognostic factors in multiple types of cancer, and the further evaluation including large cohorts of patients is required for future clinical application.

## Concluding remarks

Homo sapiens have acquired robust protective mechanisms against cancer by repressing telomerase coupled with shorter telomeres over a long evolution period. Erasing this natural barrier is required for malignant transformation of human somatic cells and in most cases, is achieved by the *TERT* gene de-repression/telomerase reactivation. Thus, a mechanistic elucidation underlying cancer-specific activation of the *TERT* transcription is of intense interest to cancer research. During the last 20 years, this issue has been extensively studied. We learnt much from the cloning of the *TERT* gene and its promoter, and identification of its key transcription factors (both positive and negative), however, the findings made in the past 6 years have significantly contributed to our in-depth understanding of telomerase biology in cancer, and the genomic aberrations have started to take center stage: the hotspot mutation at 5’ regulatory promoter region and rearrangements at the *TERT* locus are emerging as new players in activating *TERT* transcription. It should be pointed out that these genomic aberrations stimulate TERT expression by triggering massive epigenetic alterations and disrupting repressive chromatins locally. Therefore, a close genomic-epigenetic interaction is required for telomerase activation, like TPE-OLD-mediated *TERT* transcription. Interestingly, TERT promoter methylation is required for cancer cells to activate *TERT* transcription, while TERT induction in turn promotes the aberrant methylation by upregulating the expression of DNA methyltransferases, which forms a positive feedback loop [20, 23].

These new findings not only lead to profound mechanistic understanding of the cancer-specific *TERT* transcription, but also provide useful tools for clinical managements of cancer patients. However, a key challenge is how to translate these findings into a telomerase-based therapeutic strategy. The ETS family member GABPB1, required for the *TERT* transcription in TERT promoter-bearing glioblastoma cells, has been proposed as a target for glioblastoma treatment [56], but our results showed that inhibiting GABPA or GABPB1 robustly increased invasiveness of TC cells despite significant downregulation of TERT expression [59]. Likely, the GABPA/B function is context-dependent. Further studies are required for the rational development of TERT-based cancer therapeutic interventions.
